# Surface EMG signals in very late-stage of Duchenne muscular dystrophy: a case study

**DOI:** 10.1186/s12984-017-0292-4

**Published:** 2017-08-29

**Authors:** Joan Lobo-Prat, Mariska M.H.P. Janssen, Bart F.J.M. Koopman, Arno H.A. Stienen, Imelda J.M de Groot

**Affiliations:** 1 Department of Biomechanical Engineering, University of Twente, Drienerlolaan 5, Enschede, 7522 NB The Netherlands; 2 Department of Rehabilitation, Radboud University Medical Center, Reinier Postlaan 4, Nijmegen, 6500 HB The Netherlands

**Keywords:** Duchenne, Surface electromyography (sEMG), Control interface, Assistive device, Upper extremity

## Abstract

**Background:**

Robotic arm supports aim at improving the quality of life for adults with Duchenne muscular dystrophy (DMD) by augmenting their residual functional abilities. A critical component of robotic arm supports is the control interface, as is it responsible for the human-machine interaction. Our previous studies showed the feasibility of using surface electromyography (sEMG) as a control interface to operate robotic arm supports in adults with DMD (22-24 years-old). However, in the biomedical engineering community there is an often raised skepticism on whether adults with DMD at the last stage of their disease have sEMG signals that can be measured and used for control.

**Findings:**

In this study sEMG signals from Biceps and Triceps Brachii muscles were measured for the first time in a 37 year-old man with DMD (Brooke 6) that lost his arm function 15 years ago. The sEMG signals were measured during maximal and sub-maximal voluntary isometric contractions and evaluated in terms of signal-to-noise ratio and co-activation ratio. Beyond the profound deterioration of the muscles, we found that sEMG signals from both Biceps and Triceps muscles were measurable in this individual, although with a maximum signal amplitude 100 times lower compared to sEMG from healthy subjects. The participant was able to voluntarily modulate the required level of muscle activation during the sub-maximal voluntary isometric contractions. Despite the low sEMG amplitude and a considerable level of muscle co-activation, simulations of an elbow orthosis using the measured sEMG as driving signal indicated that the sEMG signals of the participant had the potential to provide control of elbow movements.

**Conclusions:**

To the best of our knowledge this is the first time that sEMG signals from a man with DMD at the last-stage of the disease were measured, analyzed and reported. These findings offer promising perspectives to the use of sEMG as an intuitive and natural control interface for robotic arm supports in adults with DMD until the last stage of the disease.

**Electronic supplementary material:**

The online version of this article (doi:10.1186/s12984-017-0292-4) contains supplementary material, which is available to authorized users.

## Introduction

People with Duchenne muscular dystrophy (DMD) lose independent ambulation by the age of ten years, followed by the development of scoliosis and loss of upper extremity function during their teens, and develop severe cardiomyopathies and respiratory problems during their twenties [1]. Life expectancy of people with DMD has substantially improved over the last five decades, due to improvements in care, drugs, and the introduction of home care technology, such as artificial ventilators [2]. As a result, there is currently a considerable group of adults with DMD living with severe physical impairments and a strong dependency on care up to their 30’s [3].

Several arm supports that compensate the weight of the arms are commercially available and have shown an increase of independence and quality of life for teenagers with DMD [4]. However, in adults with DMD, the decrease of muscle force combined with an increase of passive joint-stiffness [5], reduces the effectiveness of current arm supports [6, 7]. More advanced robotic arm supports can provide extra assistance, and have the potential to enable adults with DMD to continue performing activities of daily living, increasing their independence and participation in social activities.

In order to operate robotic arm supports, the user needs to communicate his motion intention to the device through a control interface. Currently, the only control interface available for adults with DMD are hand joysticks and switches, which are used to control wheelchairs and external robotic arms. We consider that the use of control interfaces that detect the motion intention from physiological signals that are implicitly related to the supported motion can result in a more natural and intuitive interaction with the robotic arm support. In this direction, we have developed and evaluated force and surface electromyography (sEMG) based control interfaces [8, 9].

The ability for adults with DMD to use sEMG-based control, a well-established control interface in upper-extremity prosthetics [10], depends on the availability and quality of their sEMG signals. EMG signals have been used for decades in DMD patients for diagnosis and carrier detection [11]. These studies are mostly based on invasive needle EMG recordings. Studies with sEMG in boys and men with Duchenne are less common and most of them report measurements of lower extremity, facial or oral muscles [12–14]. From a comprehensive literature review we found that Priez et. al. [15], Bowen et. al [16], Kumangai and Yamada [17], Fascarelli et al. [18], Lobo-Prat et al. [9], and Janssen et al. [19] measured sEMG signals from upper extremity muscles in subjects with DMD aged between 5 to 24 years. To the best of our knowledge there are no published studies that report the measurement of upper-extremity sEMG signals in men with DMD older than 24 years, which is the period when robotic arm supports are most needed.

In a previous study [9], we showed that both sEMG- and force-based control interfaces were feasible solutions for the control of elbow movements in adults with DMD (22-24 years-old). Force-based control was experienced as fatiguing by all participants, a fact that indicates that sEMG-based control is probably the only viable interface for adults with DMD at the last stage of the disease. However, in the biomedical engineering community there is an often raised skepticism over adults with DMD at the last stage of their disease (Brooke score 6) having sEMG signals that can be measured and used for control. As a consequence, development of assistive devices for this group of patients is getting low attention. We think that this skepticism might be based on a wrong pre-conception and that it is thus important to investigate if sEMG signals from upper-extremity muscles of men with DMD at the last stage of the disease are measurable and can be used for control.

DMD patients at the last stage of their disease are very rare and getting them involved in any study is difficult and delicate, because they easily get overwhelmed by the exercises. As a consequence, conducting tests with even just a few of these subjects is very unlikely and general conclusions will have to be drawn from a number of independent studies. We were able to get the kind collaboration of a 37 year-old man with DMD for this study to evaluate his sEMG signals from upper-extremity biceps and triceps muscles. Albeit results from only one subject are insufficient to draw general conclusions, they are relevant to be communicated because of their exceptional nature and will encourage similar studies.

While we hypothesized that the neural activation of the muscle is still measurable in men with DMD that have lost their arm function long time ago (DMD is a disease affecting the contraction of the muscle cells only), we expected their sEMG signals to have a much lower amplitude than in the case of healthy individuals: the infiltration of fatty and connective tissue in the muscle is known to increase the electrical impedance [20]. The quality of the sEMG signals was evaluated in terms of signal-to-noise ratio (SNR) and co-activation ratio (CAR). Additionally, we evaluated the feasibility of decoding the user’s movement intention from the measured sEMG simulating a sEMG-controlled elbow orthosis.

## Methods

### Participant

A 37 year-old man with DMD participated in this study. The participant was classified according to the Brooke upper extremity function scale [21] with a score of 6 (i.e. no arm/hand function was left) and lost his arm function long time ago: shoulder movements more than 20 years ago, and elbow flexion-extension more than 15 years ago. He was able to control an electric wheelchair with a highly sensitive joystick and several push buttons that were operated using residual motion of the fingers of both hands. No other functional tasks were possible with his arms or hands. The participant also presented joint contractures caused by the disuse of the arms, which severely limited the range-of-motion of all the upper-extremity joints.

### Signal acquisition and processing

The sEMG signals were measured from the Biceps Brachii and Triceps Brachii muscles of the right arm, which originally was the dominant arm. Two 99.9% Ag parallel bars electrodes (contact: 10 mm x 1 mm each) spaced 10 mm apart (Bagnoli DE-2.1. Delsys; Boston, Massachusetts) were placed in parallel with the muscle fibers following to the SENIAM recommendations [22] and manual muscle exploration, after the skin was shaved and scrubbed clean. The signals were amplified with a Delsys Bagnoli-16 Main Amplifier and Conditioning Unit (Delsys; Boston, Massachusetts) with a bandwidth of 20 to 450 Hz and a gain of 1000.

The sEMG signals were measured with a data acquisition card with a sampling frequency of 1 kHz and 16 bit resolution. The offset of the raw sEMG signals was removed on-line using a fourth-order Butterworth high-pass filter with a cutoff frequency of 20 Hz. Subsequently the signals were full-wave rectified and smoothed using a second-order low-pass Butterworth filter with a cutoff frequency of 1 Hz to obtain the signal envelope. The envelopes were used for the visual feedback during the maximal voluntary isometric contractions (MVIC) and sub-maximal voluntary isometric contractions (SVIC), for the analysis of the CAR, and for the simulation of the elbow orthosis.

### Measurement protocol

The participant was asked to perform a series of MVICs and SVICs with his biceps and triceps muscles. The MVIC were measured to investigate the maximal signal amplitude, and the SVIC were measured to investigate if the participant could voluntarily modulate the intensity of the muscle activation. Since the participant had not been able to voluntarily flex and extend his elbow joint for a long time, the examiner passively moved his elbow joint and applied pressure against the intended movement to familiarize the subject with the task. For the MVIC measurements the researcher asked the participant to maximally flex and extend his elbow during three seconds, three times for each muscle. For the SVIC measurements the subject was asked to reach three activation levels (20%, 40% and 80% of MVIC) during three seconds for each muscle. Note that the actual activation levels achieved by the participant were slightly different from these target levels (see “Results” section). Having three activation levels, only one run for each level could be performed to avoid overwhelming the participant with too long a session. All measurements were guided with real-time visual feedback of the sEMG envelopes displayed on a computer screen. For the SVICs the researcher pointed at three levels on the computer screen used for visual feedback. Between each muscle contraction the participant rested during five to ten seconds and between each series of MVIC or SVIC tasks the participant rested during five to ten minutes.

### Data analysis

The quality of the sEMG signals was evaluated in terms of the SNR and the CAR. Additionally, the measured sEMG signals were used to drive a simulated elbow orthosis to evaluate the feasibility of using the sEMG signals for control purposes.

#### Signal-to-noise ratio

The SNR of the biceps (*S* *N* *R*_*b*_) and of the triceps (*S* *N* *R*_*t*_) were calculated by taking the ratio between the power of the root-mean-squared amplitude of the raw sEMG signal (*R* *M* *S*_*b*_, *R* *M* *S*_*t*_) during the three MVIC and SVIC measurements, and the power of the RMS amplitude of the raw sEMG signal during a resting period, which represented the noise level (*R* *M* *S*_*nb*_, *R* *M* *S*_*nt*_):

1 $$ {SNR}_{b}=\left(\frac{RMS_{b}}{RMS_{nb}}\right)^{2},\quad {SNR}_{t}=\left(\frac{RMS_{t}}{RMS_{nt}}\right)^{2}.  $$

The SNR of the biceps (${SNR}_{b_{dB}}$) and of the triceps (${SNR}_{t_{dB}}$) expressed in decibels (dB) was calculated using:

2 $$ {}{SNR}_{b_{dB}}\,=\,10\!\log_{10}\!\left(\!\frac{RMS_{b}}{RMS_{nb}}\!\right)^{2}\!,\ \ {SNR}_{t_{dB}}\,=\,10\!\log_{10}\!\left(\!\frac{RMS_{t}}{RMS_{nt}}\!\right)^{2}\!.  $$

The highest RMS value found within the three repetitions of the MVIC measurement was used to normalize the EMG signals of the biceps and triceps muscles for the CAR calculation.

#### Co-activation Ratio

Similarly to the SNR, we evaluated the involuntary activation of the antagonistic muscle by calculating the CAR of the biceps (*C* *A* *R*_*b*_) and triceps (*C* *A* *R*_*t*_). The CAR was defined as the ratio between the RMS amplitude of the normalized sEMG signal envelope of the agonist muscle (*R* *M* *S*_*Nb*_, *R* *M* *S*_*Nt*_), and the RMS amplitude of the normalized sEMG signal envelope of the antagonist muscle (*R* *M* *S*_*Nt*_, *R* *M* *S*_*Nb*_) during the three MVIC and SVIC measurements (Eq. 3). A high CAR value indicates a high level of co-activation while a low CAR value indicates that a low level co-activation is present.

3 $$ {CAR}_{b}=\frac{RMS_{Nt}}{RMS_{Nb}},\quad {CAR}_{t}=\frac{RMS_{Nb}}{RMS_{Nt}}  $$

The normalized signal envelopes of the biceps (*R* *M* *S*_*Nb*_) and triceps (*R* *M* *S*_*Nt*_) were calculated by dividing the RMS value of the signal envelope (*R* *M* *S*_*Eb*_, *R* *M* *S*_*Et*_) by the RMS value of the signal envelope of the MVIC (${RMS}_{Eb_{MVIC}}$, ${RMS}_{Et_{MVIC}}$; Eq. 4). Note that the RMS value of the noise envelope (*R* *M* *S*_*Enb*_, *R* *M* *S*_*Ent*_) was subtracted from signal envelopes before calculating *R* *M* *S*_*Nb*_ and *R* *M* *S*_*Nt*_.

4 $$ \begin{aligned} {RMS}_{Nb}=\frac{({RMS}_{Eb} - {RMS}_{Enb})}{{RMS}_{{Eb}_{MVIC}}- {RMS}_{Enb})}, \\ {RMS}_{Nt}=\frac{({RMS}_{Et} - {RMS}_{Ent})}{{RMS}_{{Et}_{MVIC}}- {RMS}_{Ent})} \end{aligned}  $$

#### Elbow orthosis simulation

In order to evaluate the feasibility of using the measured sEMG signals for control purposes, we performed an off-line simulation (i.e. open-loop control) of a sEMG-controlled elbow orthosis. This method was chosen because the high intrinsic stiffness and contractures present in the elbow joint of the participant prevented the use of a real robotic elbow orthoses.

The sEMG-based control method implemented in the simulation and described in this subsection was the same used in our previous study [9] were elbow movements of adults with DMD were supported with an elbow orthosis. An estimation of the elbow torque ($\hat {\tau }_{e}$) was obtained by multiplying the signal envelops of the biceps and triceps sEMG signals (*E*_*b*_, *E*_*t*_) by a mapping gain (*K*_*b*_, *K*_*t*_) and subsequently subtracting the triceps signal from the biceps signal:

5 $$ \hat{\tau}_{e}=E_{b} K_{b}-E_{t} K_{t}.  $$

**Fig. 1:**
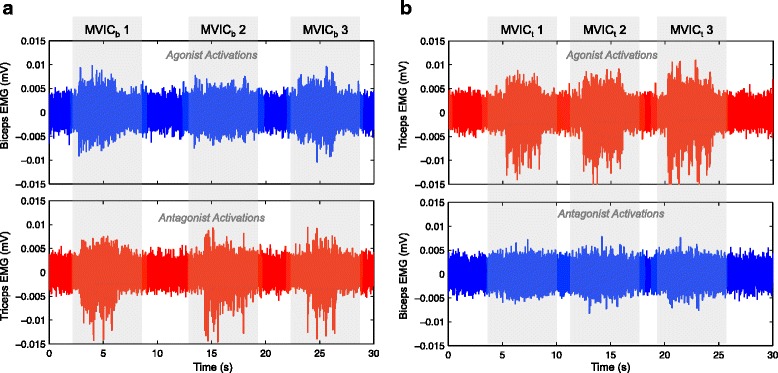
Raw sEMG signals during three maximal voluntary isometric contractions (MVIC) of biceps and triceps muscles. **a** Agonist activation of biceps in *blue*; signal of antagonist muscle (triceps) in *red*. RMS values for each MVIC of the biceps: *M* *V* *I* *C*_*b*_1=0.0021 mV, *M* *V* *I* *C*_*b*_2=0.0016 mV, *M* *V* *I* *C*_*b*_3=0.0019 mV. **b** Agonist activation of triceps in *red*; signal of antagonist muscle (biceps) in *blue*. RMS values for each MVIC of the triceps: *M* *V* *I* *C*_*t*_1=0.0024 mV, *M* *V* *I* *C*_*t*_2=0.0026 mV, *M* *V* *I* *C*_*t*_3=0.0025 mV

Note that a fix offset resulting from the sEMG signal noise was removed from the signal envelopes to obtain *E*_*b*_ and *E*_*t*_. The offset value was calculated by taking the mean value of the signal envelope during three seconds while the participant was relaxed. For the simulation, the signals measured during the MVIC of the biceps and of the triceps (shown in Fig. 1) were concatenated and used as *E*_*b*_ and *E*_*t*_. The mapping gains *K*_*b*_=2 Nm/mV and *K*_*t*_=0.72 Nm/mV were chosen to properly distinguish biceps from triceps activation and to obtain a symmetric elbow flexion-extension movement.

The estimated elbow torque was then used as input for an admittance model that rendered the dynamics of a mass-damper system and had as output the elbow angle (*θ*_*e*_):

6 $$ \theta_{e} (s)=\frac{1}{(I_{v} s^{2}+B_{v} s)}\hat{\tau}_{e} (s),  $$

where *I*_*v*_ and *B*_*v*_ represent the virtual mass and damping parameters of the admittance model respectively, and *s* is the Laplace transform variable. Note that in a real elbow orthosis, the elbow angle (*θ*_*e*_; or the angular velocity) resulting from Eq. (6) would be used as reference signal for a low-level position (or velocity) controller of the elbow orthosis as in [9]. The simulation was carried out with *I*_*v*_=4·10^−3^ kg m^2^ and *B*_*v*_=1·10^−3^ Nm s/rad respectively, which resulted in a motion close to the natural range of the elbow joint. In a real elbow orthosis the interface dynamics and the mapping gains would be chosen to the convenience of the user.

## Results

### Maximal voluntary isometric contractions (MVIC)

The raw sEMG signals of the MVICs during three seconds presented maximum amplitudes of 0.01 and 0.015 mV for biceps and triceps muscles respectively (Fig. 1). The RMS values of the sEMG signals of the agonist muscle were higher for the triceps (*M* *V* *I* *C*_*t*_ in Fig. 1) than for the biceps (*M* *V* *I* *C*_*b*_ in Fig. 1) in all three repetitions, with an average value of 0.0025 mV for the triceps and 0.0019 mV for the biceps. Also, the mean SNR (Fig. 4 a) of the triceps during MVIC was double than that of the biceps (*S* *N* *R*_*t*_:4.16±0.5 (12.4±1 dB); *S* *N* *R*_*b*_:2.17±0.5 (6.7±2 dB); Fig. 4 a and b). Both RMS and SNR values were lower for the biceps than for the triceps which could indicate a higher muscle atrophy of the biceps (infiltration of fatty and connective tissue in the muscle, which degrades the sEMG signal).

Mean CAR values for both muscles during the MVICs were 0.55±0.2 for the biceps and 0.44±0.2 for the triceps (*C* *A* *R*_*b*_ and *C* *A* *R*_*t*_ respectively in Eq. 3), which indicated profound co-activation of the antagonist muscle, even more so for the biceps than for the triceps (Fig. 4 c).

### Sub-maximal voluntary isometric contractions (SVIC)

**Fig. 2:**
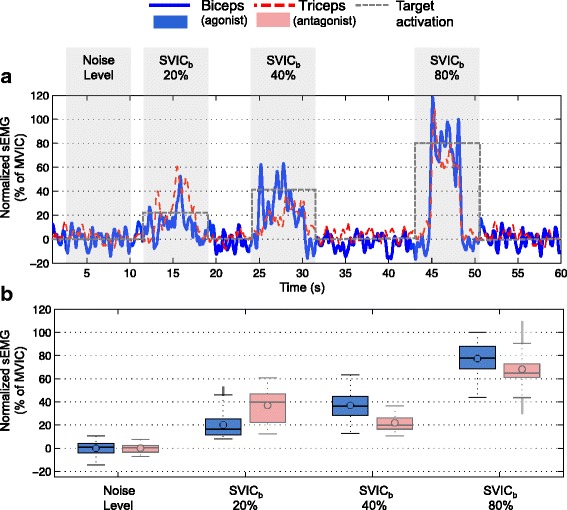
Envelope of the sEMG signals during sub-maximal voluntary isometric contractions of biceps muscle. **a** Envelope of the sEMG signals measured during the 20%, 40% and 80% *S* *V* *I* *C*_*b*_ of the biceps muscle in *blue*. Envelope of the antagonist muscle (triceps) in *dashed red*. **b** Boxplots of the 3000 data points (i.e. 3 seconds) measured for each of the SVIC levels shown in A. In *blue* the boxplots of the biceps sEMG signals and in *faded red* the boxplots of the antagonist muscle (triceps). Note that the noise level of the sEMG signal during relaxation is also shown

**Fig. 3:**
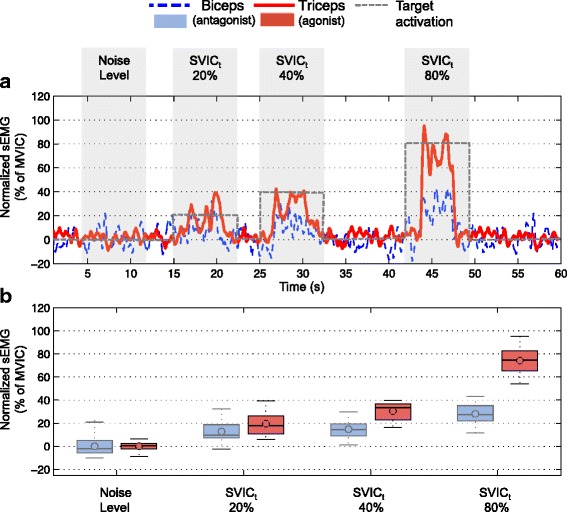
Envelope of the sEMG signals during sub-maximal voluntary isometric contractions of triceps muscle. **a** Envelope of the sEMG signals measured during the 20%, 40% and 80% *S* *V* *I* *C*_*t*_ of the triceps muscle in *red*. Envelope of the antagonist muscle (biceps) in *dashed blue*. **b** Boxplots of the 3000 data points (i.e. 3 seconds) measured for each of the SVIC levels shown in A. In *red* the boxplots of the triceps sEMG signals and in *faded blue* the boxplots of the antagonist muscle (biceps). Note that the noise level of the sEMG signal during relaxation is also shown

**Fig. 4:**
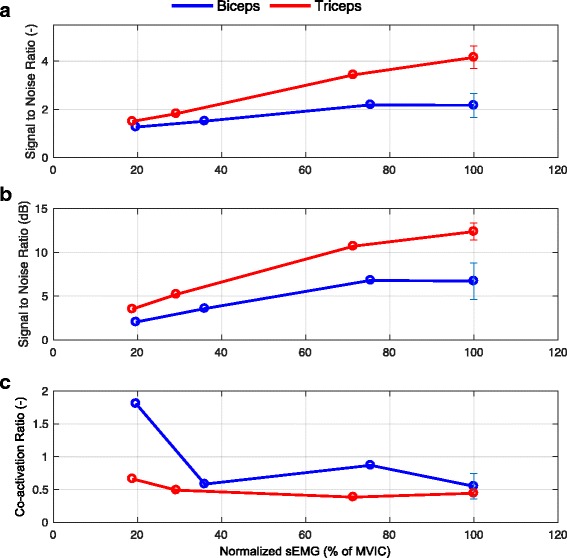
Signal-to-noise ratio (SNR) and co-activation ratio (CAR) for the biceps and triceps sEMG signals as function of activation level. **a** Signal-to-noise ratios of the biceps (*blue*) and triceps (*red*) sEMG signals during the three SVIC and MVIC. **b** Signal-to-noise ratios of the biceps (*blue*) and triceps (*red*) sEMG signals during the three SVIC and MVIC expressed in decibels (dB). **c** Co-activation ratios of the biceps (*blue*) and triceps (*red*) sEMG signals during the three SVIC and MVIC. Note that the error bar is only shown for the MVIC as this measurement was repeated three times

When performing the SVICs, the participant was able to generate sEMG signals that followed the level of effort demanded by the task (Figs. 2 and 3), indicating that the participant could voluntarily modulate the required level of muscle activation. The SNR of the SVICs increased for both biceps and triceps muscles with the increase of SVIC level (Fig. 4 a), as expected because of the raise in signal amplitude. In agreement with the results of the MVICs, the SNRs of the triceps muscle were higher than the ones of the biceps muscle for all conditions.

The CARs of the biceps muscle were higher than the CARs of the triceps muscle for all levels of SVIC (Fig. 4 c). While the CARs of the triceps muscle presented values close to 0.5 for all SVICs (with the exception of the 20% SVIC level) the CARs of the biceps muscle presented more variability over activation levels: the CAR of the 20% SVIC level was above 1.5, the CAR of the 40% SVIC level and of 100% was close to 0.5, and the CAR of the 80% SVIC level was close to 1.

### Elbow orthosis simulation

**Fig. 5:**
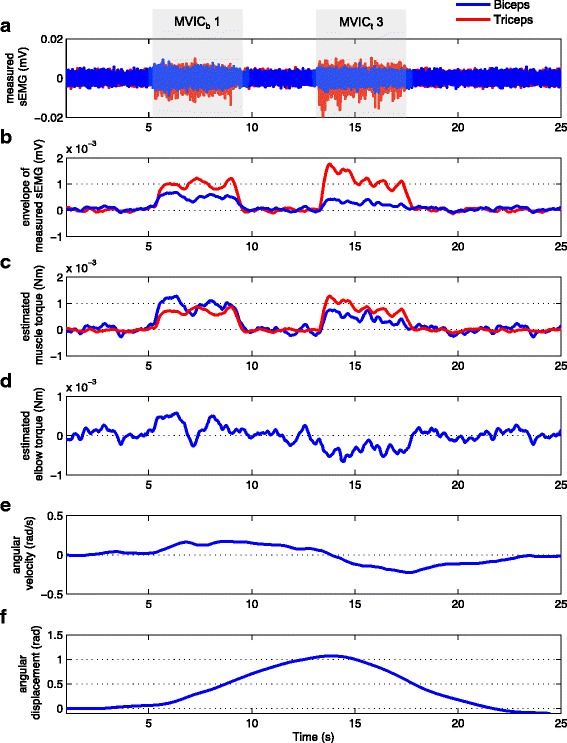
Simulation of the sEMG-controlled elbow orthosis. **a** Raw sEMG signals used as input for the simulation. Specifically the first (*M* *V* *I* *C*_*b*_1) and third (*M* *V* *I* *C*_*t*_3) MVIC attempts of the biceps (*blue*) and triceps (*red*). **b** Envelopes of the raw sEMG signals of the biceps (*blue*) and triceps (*red*). **c** Estimated muscle torque of the biceps (*blue*) and triceps (*red*) obtained by multiplying the envelopes multiplying by the mapping gains *K*_*b*_ and *K*_*t*_. **d** Estimated elbow torque calculated by subtracting the estimated triceps torque from the estimated biceps torque (Eq. 5). **e** Angular velocity resulting from the admittance model (Eq. 6). **f** Elbow angle displacement resulting from the integral of the angular velocity (Eq. 6)

Figure 5 a and b show the raw and the envelope of the sEMG signals respectively used as input for the simulation. Figure 5 c shows the estimated muscle torque calculated by multiplying the signal envelope with the mapping gains *K*_*b*_ and *K*_*t*_. Note that after applying the mapping gains, the biceps signal is larger than the triceps signal for the first half of the movement and the triceps signals is larger then the biceps signal for the second half of the movement. Figure 5 d shows the estimated elbow torque resulting from the difference between the biceps and triceps muscle torques. Figure 5 e presents the output of the admittance controller and shows that the simulated system responded with a positive angular velocity during the first part of the movement (biceps activation) and a negative angular velocity during the second part (triceps activation). The integral of this angular velocity is shown in Fig. 5 f, which indicates that the simulated orthosis would successfully support an elbow flexion and extension movement of 1 rad in 20 s with a maximum speed of ±0.2 rad/s under the control of the realistic sEMG signals shown in Fig. 5 a. Note that the apparent delay between the muscle activation signal and the initiation of the movement is due to the phase lag of the second order dynamics of the admittance model with low mass and damping Eq. (6). In our previous studies [9, 23], we have seen that these kind of interaction dynamics are usable by adults with DMD.

Figures 6 and 7 in Additional file 5 show the results obtained with the same elbow orthosis model but using all three repetitions of the MVICs and of the SVICs as input signal. These results indicate that it is possible to distinguish three flexion movements following the three MVICs or SVICs of the biceps, and three extension movements following the three MVICs or SVICs of the triceps. Differences in angular velocity and displacement of the movements are due to differences in amplitude and duration of each of the MVICs or SVICs signals. Additional files 1, 2, 3, and 4 contain the datasets of sEMG signals used in this study.

## Discussion

Our results revealed the profound deterioration of the upper-extremity muscles of the 37-year-old man with DMD. The maximum amplitudes of the sEMG signals of the participant were 100 times lower than those typical of healthy individuals (i.e. our measurements of 0.01 mV vs. the 1 mV measured in healthy individuals [24]). These low signal amplitudes implied low SNRs. We also found profound involuntary activation of the antagonistic muscle as revealed by the measured CARs, which could be caused by the disuse of the arms. Note that arm immobilizations of 12 h are sufficient to significantly reduce motor performance in healthy subjects [25]. Probably with practice the participant would learn to isolate better the activation of the muscles.

Despite the deterioration of the muscles, we cannot underestimate the fact that sEMG signals from the biceps and triceps muscles were still measurable in a 37-year-old man with DMD that presented considerable muscle deterioration since 20 years ago and completely lost his arm function 15 years ago. Our results also indicate that the participant was able to adjust his voluntary isometric contractions as demanded by the exercises.

The relevance of detecting measurable upper-extremity sEMG signals in adults with DMD at the late-stage of the disease extends beyond its clinical interest. sEMG signals can be used to detect the users’ motion intention and control rehabilitation or assistive devices, which have the potential to delay the disease progression and increase the quality of life for men with DMD [26, 27]. On this regard, our simulation, which used the measured sEMG signals as input, suggests that, if the high degree of joint stiffness and contractures were not present, the participant could control an active elbow orthosis to perform flexion-extension movements with the same proportional sEMG-based control method used in our previous study [9]. The angular velocity and displacement obtained by the simulation indicated that it is possible to detect the elbow flexion/extension movement intention of the user from the measured sEMG of the biceps and the triceps muscle. Nevertheless, these results need to be regarded with caution since we did not test the performance of the sEMG-based control using a real system.

Currently, the use of robotic elbow orthoses in adults with DMD in the last stage of the disease is not an option due to the high intrinsic stiffness and joint contractures. However, the use of arm supports, would allow people with DMD to keep using their arms and therefore contribute to the delay of their functional deterioration. We expect that in the future, boys and men with DMD will use arm supports from an early age, which would preserve the range of motion of their joints and potentially benefit from the use of sEMG-controlled arm supports until the last stage of the disease.

## Conclusions

The results of the present case study indicate that sEMG signals from the biceps and triceps muscles were very deteriorated but still measurable in a 37 year-old man with DMD that lost his arm function several years ago. Also, the participant was able to adjust his muscle activation level as demanded by the SVIC tasks. To the best of our knowledge this is the first time that sEMG signals from a man with DMD at the last-stage of the disease were measured and reported. Despite the muscle deterioration, the measured signals could be successfully used as input for the control of a simulated elbow orthosis. These results offer promising perspectives to the use of sEMG as an intuitive and natural control interface for assistive devices in adults with DMD until the last stage of the disease, provided that the use of assistive devices since an early stage of the disease reduce joint stiffness and contractures.

Results from only one subject are insufficient to draw general conclusions, but the difficulties to involve participants with DMD in the last stage of the disease make the inclusion of several patients in one single study highly complicated. Thus, sufficient evidence should come by integrating independent studies performed in different laboratories. We hope that the results presented in this Short Report will start breaking the current general opinion that sEMG signals are too weak in DMD patients at the last stage of the disease to be used for control, and will encourage similar studies in other parts of the world, finally leading to better assistive devices for adults with DMD.

## Additional files

Additional file 1 Raw sEMG signals during the biceps MVICs. MAT file containing two vectors of data (30001x1) of the raw sEMG signals of the biceps (EMGb) and the triceps (EMGt). Data was measured at 1 kHz. (MAT 421 kb)

Additional file 2 Raw sEMG signals during the triceps MVICs. MAT file containing two vectors of data (30001x1) of the raw sEMG signals of the biceps (EMGb) and the triceps (EMGt). Data was measured at 1 kHz. (MAT 424 kb)

Additional file 3 Raw sEMG signals during the biceps SVICs. MAT file containing two vectors of data (59806x1) of the raw sEMG signals of the biceps (EMGb) and the triceps (EMGt). Data was measured at 1 kHz. (MAT 854 kb)

Additional file 4 Raw sEMG signals during the triceps SVICs. MAT file containing two vectors of data (59706x1) of the raw sEMG signals of the biceps (EMGb) and the triceps (EMGt). Data was measured at 1 kHz. (MAT 853 kb)

Additional file 5 Additional results of the simulation of the sEMG-controlled elbow orthosis. PDF file showing two figures with additional results of the simulation of the sEMG-controlled elbow orthosis. (PDF 899 kb)

## Abbreviations

CAR: Co-activation ratio
DMD: Duchenne muscular dystrophy
MVIC: Maximal voluntary isometric contraction
RMS: Root mean squared
sEMG: surface electromyography
SNR: Signal to noise ratio
SVIC: Sub-maximal voluntary isometric contraction
