# Supplementary material for: Surface EMG signals in very late-stage of Duchenne muscular dystrophy: a case study
Source: J Neuroeng Rehabil. 2017 Aug 29;14:86. doi: 10.1186/s12984-017-0292-4 (PMC5576133; doi:10.1186/s12984-017-0292-4)
Supplement: Supplementary file 5 — Additional results of the simulation of the sEMG-controlled elbow orthosis. PDF file showing two figures with additional results of the simulation of the sEMG-controlled elbow orthosis. (PDF 899 kb) [file 12984_2017_292_MOESM5_ESM.pdf]

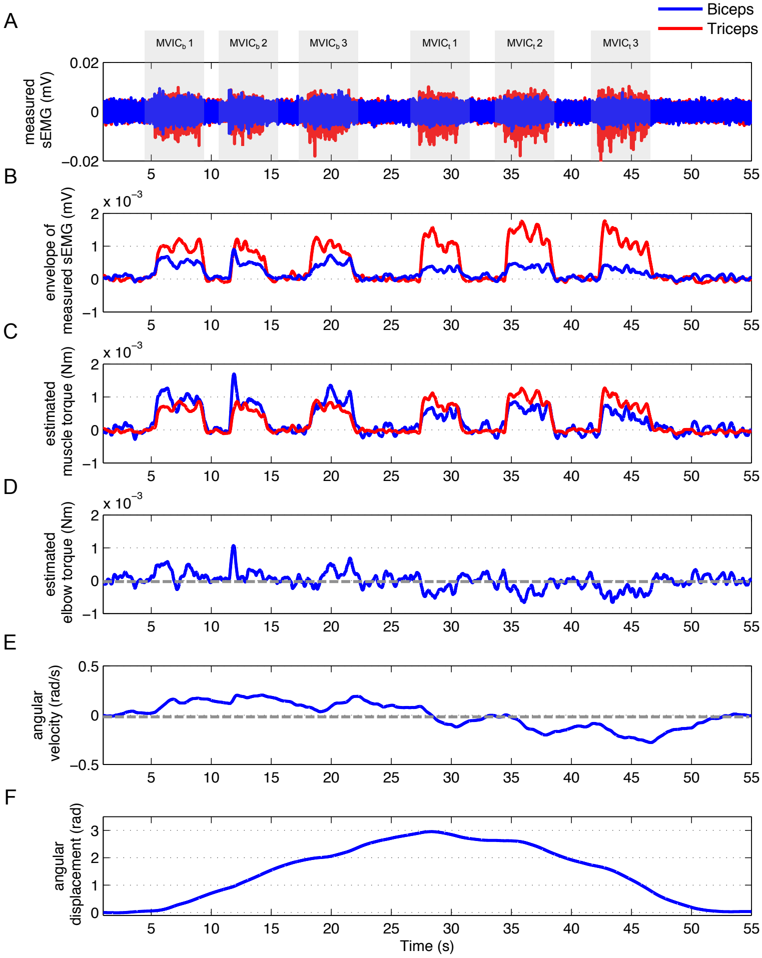

Figure 6 Additional results of the simulation of the sEMG-controlled elbow orthosis. A) Raw sEMG signals used as input for the simulation. Specifically the three MVICs of the biceps (blue; $MVIC_b1$, $MVIC_b2$, $MVIC_b3$) and the three MVICs of the triceps (red; $MVIC_t1$, $MVIC_t2$, $MVIC_t3$). **B**) Envelopes of the raw sEMG signals of the biceps (blue) and triceps (red). **C**) Estimated muscle torque of the biceps (blue) and triceps (red) obtained by multiplying the envelopes multiplying by the mapping gains K_b and K_t. **D**) Estimated elbow torque calculated by subtracting the estimated triceps torque from the estimated biceps torque (Eq 4). **E**) Angular velocity resulting from the admittance model (Eq 5). **F**) Elbow angle displacement resulting from the integral of the angular velocity (Eq 5).

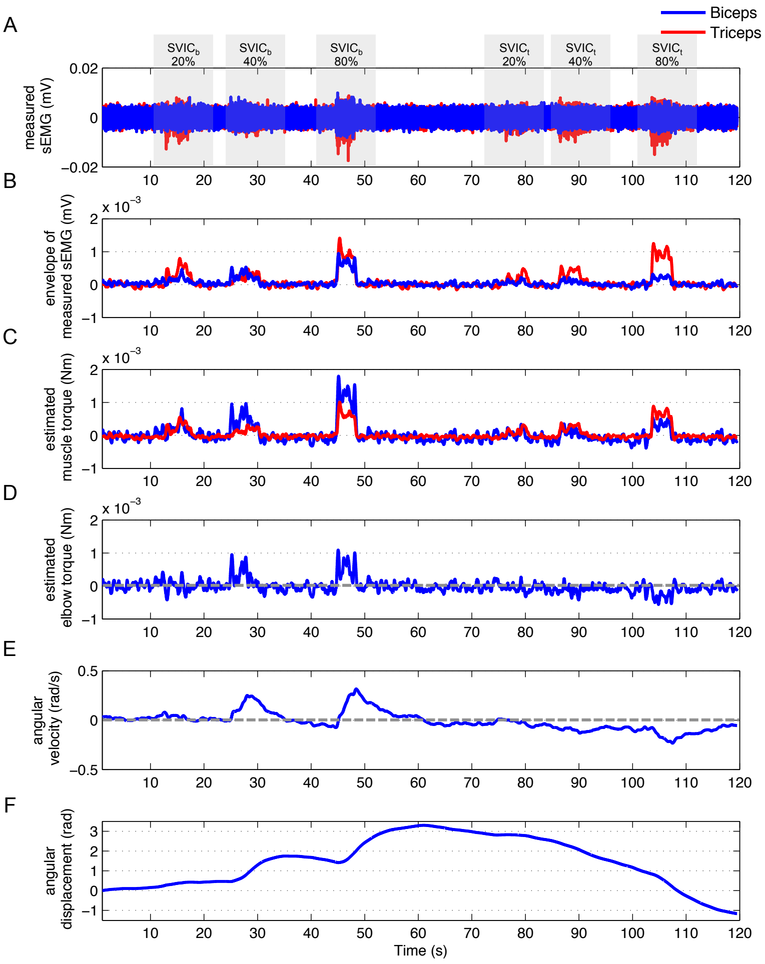

Figure 7 Additional results of the simulation of the sEMG-controlled elbow orthosis. A) Raw sEMG signals used as input for the simulation. Specifically the three SVIC of the biceps (blue; $SVIC_b$ 20%, $SVIC_b$ 40%, $SVIC_b$ 80%) and the three SVICs of the triceps (red; $SVIC_t$ 20%, $SVIC_t$ 40%, $SVIC_t$ 80%). **B**) Envelopes of the raw sEMG signals of the biceps (blue) and triceps (red). **C**) Estimated muscle torque of the biceps (blue) and triceps (red) obtained by multiplying the envelopes multiplying by the mapping gains K_b and K_t. **D**) Estimated elbow torque calculated by subtracting the estimated triceps torque from the estimated biceps torque (Eq 4). **E**) Angular velocity resulting from the admittance model (Eq 5). **F**) Elbow angle displacement resulting from the integral of the angular velocity (Eq 5).
